# Daily Head and Neck Treatment Assessment for Optimal Proton Therapy Planning Robustness

**DOI:** 10.3390/cancers15143719

**Published:** 2023-07-22

**Authors:** Leslie Chang, Sherif G. Shaaban, Emile Gogineni, Brandi Page, Harry Quon, Heng Li, Rachel Ger

**Affiliations:** 1 Department of Radiation Oncology and Molecular Radiation Sciences, Johns Hopkins University School of Medicine, Baltimore, MD 21202, USArger2@jhmi.edu (R.G.); 2 Department of Radiation Oncology, Ohio State University, Columbus, OH 43210, USA

**Keywords:** head and neck cancer, proton, particle radiotherapy, quality assurance, robustness optimization

## Abstract

**Simple Summary:**

Proton therapy requires robust optimization to ensure patients receive target coverage and spare normal tissues. However, validation of setup variables has not been studied in patients receiving head and neck proton therapy. The aim of our retrospective study was to evaluate the absolute fractional deviation for dose and volume treatment metrics using 3 mm vs. 5 mm setup robustness in patients treated with head and neck proton therapy. We found that deviations from planning clinical target volumes were greater using 3 mm as compared to 5 mm setup robustness. In addition, variation was greater for patients with primary and secondary clinical targets as compared to primary targets alone. However, the average fractional deviation was less than 1% in primary targets and up to 2% in secondary targets using 3 mm setup uncertainty. We recommend robustness optimization using 3 mm setup uncertainty with daily CBCT for patients being treated with proton head and neck therapy.

**Abstract:**

Robust optimization in proton therapy ensures adequate target coverage; however, validation of fractional plan quality and setup uncertainty in patients has not been performed. We aimed to assess plan robustness on delivered head and neck proton plans classified into two categories: (1) primary only (PO) and (2) primary and neck nodal (PNN) coverage. Registration at the machine was utilized for daily CBCT to generate a synthetic CT. The dose for the clinical target volume (CTV) and organs at risk (OAR) was compared to the expected robustness bands using 3.5% range uncertainty and 3 mm vs. 5 mm setup uncertainty. The fractional deviation was defined as D95% and V100% outside of uncertainty constraints. About 203 daily fractions from 6 patients were included for analysis. The percentage of fractions that exceeded robustness calculations was greater in 3 mm as compared to 5 mm setup uncertainty for both CTV and OAR volumes. PO plans had clinically insignificant average fractional deviation, less than 1%, in delivered D95% and V100%. In comparison, PNN plans had up to 2.2% average fractional deviation in delivered V100% using 3 mm robustness. Given the need to balance dose accuracy with OAR sparing, we recommend the utilization of 3 mm setup uncertainty as an acceptable simulation of the dose delivered.

## 1. Introduction

Radiation therapy is an effective treatment modality to definitively manage early-stage head and neck cancer as well as improve locoregional control in the adjuvant setting [1,2]. Intensity-modulated proton therapy (IMPT) has unique properties as compared to traditional photon radiation therapy and may be clinically advantageous in treating head and neck cancer as it combines the conformality of intensity-modulated radiation therapy and the lower integral dose to normal tissues. This is particularly important for many patients with head and neck cancer who have excellent long-term disease outcomes but are in close proximity to critical organs at risk [3]. The physical advantage of proton dosimetry is due to the Bragg peak phenomenon, resulting in a sharp increase in the dose deposited at the end of the particle range and the absence of an exit dose beyond the target. Due to sharp distal dose fall-off, proton treatment has increased sensitivity to organ motion, setup and anatomical variations and calculation accuracy from computed tomography (CT) images and conversion of Hounsfield units to stopping power, which need to be accounted for during the treatment planning process [4,5].

Owing to the uncertainty in the localization of the Bragg peak, it is important to improve quality assurance to account for technical uncertainty and ensure the integrity of the treatment. During IMPT planning in head and neck patients, beam geometry is adjusted to minimize dose heterogeneity, such as avoiding surgical or dental hardware [6], choosing short beam paths and minimizing beams traversing hollow organs [7]. In-room or gantry-mounted, cone beam CT (CBCT) has been used to obtain information on daily patient positioning and anatomical variations. However, our understanding of daily setup, range and anatomical uncertainty is typically derived from simulations of delivered dose and has been found that coverage of the clinical target volume (CTV) can significantly decline due to the uncertainty of inter- and intra-fractional dose distribution [8,9,10]. Clinically, we assess plan optimization by evaluating the coverage of the CTV under different robustness scenarios. Therefore, robust optimization typically accounts for worst-case scenarios of range uncertainty and daily variations in setup in order to ensure target coverage [11,12,13].

In practice, setup uncertainty can vary institutionally, typically ranging from 3 to 5 mm standard deviation in all directions. ICRU 93 gives recommendations for the prescription, recording and reporting of ion beam therapy and suggests patient fixation and setup as a small (below 3%) uncertainty; however, it recommends the suitability of margins be validated for clinical use [14]. A better understanding of the optimal robustness in daily setup variation will allow providers to deliver a clinically meaningful treatment and maximize normal tissue sparing. Direct dose calculation on daily CBCT is not recommended due to poor image quality, which limits the accuracy of proton dose calculations [15]. Several groups have demonstrated that CBCT can be used for reliable proton dose calculation by utilizing a deformable image registration of the planning CT to the daily CBCT to develop a synthetic CT [16,17,18]. We aimed to provide recommendations for proton robustness criteria by performing dose recalculations using daily synthetic computed tomography (CT) to evaluate target and organs at-risk coverage using 3 mm vs. 5 mm setup uncertainty.

## 2. Materials and Methods

### 2.1. Study Participants

All patients treated for head and neck cancer at the Johns Hopkins Proton Therapy Center between November 2019 and February 2023 and who had completed treatment were eligible for inclusion. To examine the impact of setup uncertainty for proper robustness criteria, our inclusion criteria focused on patients who completed their treatment course with the same plan and setup immobilization. Patients eligible for inclusion had a full field of view CBCT for more than 75% of their daily fractions. Patients were excluded if they required an adaptive proton plan or were only treated with protons for part of their treatment course. This work was conducted with a waiver of informed consent under Johns Hopkins Institutional Review Board approval. Patients were immobilized using the Civco Proform Proton Thermoplastic mask (Civico, Coralville, IA, USA). All patients were treated using a ProBeat compact-gantry pencil beam scanning proton therapy system (Hitachi, Tokyo, Japan) with a gantry-mounted CBCT. Plans were created using 2 to 5 beams with a 4 cm range shifter using Raystation (version 10a; RaySearch Laboratories, Stockholm, Sweden). Using CTV-based treatment planning, plans were optimized robustly with 3 mm setup uncertainty and 3.5% range uncertainty. Using individual-field simultaneous optimization, simultaneous optimization of multiple fields was performed using a split target volume technique [19]. The final dose was computed using Monte Carlo with 0.5% statistical uncertainty. All doses reported are using a constant relative biological effectiveness of 1.1.

### 2.2. Robustness Analysis

Patients were classified into two categories: (1) primary only (PO) coverage without locoregional neck nodal treatment and (2) primary and neck nodal (PNN) locoregional coverage. Patients with multiple nodal volumes had all volumes assessed as a secondary target. For each patient, daily shifts at the treatment machine (superior–inferior, left–right, anterior–posterior, roll, pitch and yaw) from daily CBCT images were recorded. Registration used at the machine was utilized for each daily CBCT to generate a synthetic CT using a previously validated method [16].

We used the proton gantry-mounted CBCT (Hitachi ProBeat, Hitachi Ltd, Santa Clara, USA), which has a maximum field of view (FOV) of 38 cm × 38 cm × 29 cm. The images were acquired in full-scan (179.9–180°) mode at 100 kVp. The CBCT was aligned to the planned CT scan based on the image transformation extracted from the time of treatment. Next, the planning CT was deformed to the CBCT within the RayStation 10A SP1 (RaySearch, Stockholm SE) treatment planning system (TPS) using the built-in anatomically constrained deformation algorithm (ANACONDA), which combines anatomical information and intensities and has been previously validated [20]. We employed default deformable image registration (DIR) settings, which performed well based on a manual review of the deformed images. The initial and final DIR grid resolution sizes were 5 mm isotropic and 2.5 mm isotropic, respectively. Initial and final Gaussian smoothing sigma were 2 and 0.33. The initial and final grid regularization weight was 400. The maximum number of iterations per resolution level was 1000.

Once the planning CT was deformably registered to the CBCT, it was resampled in the CBCT frame of reference through an in-house Python script within Raystation, thus creating a synthetic CT. The CTVs and organs at risk (OARs) were manually edited by two physicians to maintain fidelity. We did not find large changes in the lumen filling of organs like the esophagus and trachea; however, our patient population did not have significant anatomical change as patients requiring an adaptive plan were excluded from analysis in order to best evaluate the daily setup uncertainty. The original isocenter and treatment fields were transferred onto the synthetic CT using the rigid alignment defined at the time of treatment, and plan dose was re-computed on the synthetic CT using the Monte Carlo algorithm. CTV-based planning is used with robust optimization to analyze the coverage of the CTV under different robustness scenarios [13]. Each patient’s plan was calculated using two scenarios: (1) 3.5% range uncertainty and 3 mm setup uncertainty and (2) 3.5% range uncertainty and 5 mm setup uncertainty. For each of these scenarios, a band of expected doses was created and the maximum and minimum from these bands were selected to determine the range.

For each fraction, the dose computed on the synthetic CT was compared to the range expected for the scenarios of 3 mm and 5 mm setup uncertainty. Target coverage was assessed by the dose received to 95% of the target in cGy (D95%) and the percentage of volume receiving 100% of the dose (V100%). The maximum dose for the brainstem, eyes, optic chiasm, optic nerves, spinal cord and mandible was assessed, and the mean dose for the parotids, cochlea, cricopharyngeus and esophagus was assessed. OARs that were relevant to the target site were analyzed for each patient. The supplementary material contains information on the number of patients assessed for each OAR (Supplementary Table S1).

For each target and OAR, the percent of fractions within the range for the 3 mm and 5 mm setup uncertainty scenarios was calculated. For targets that were outside the uncertainty range, the fractional dose deviation was calculated using Equation (1) for the D95% metric.

(1) $$Fractional\;Dose\;Deviation=\frac{Min\left(D_{fx}-D_{Rmax},D_{Rmin}-D_{fx}\right)}{D_{Rx}}\times 100$$

where *D_fx_* is the dose for the given fraction, *D_Rmax_* is the maximum dose from the range for the given scenario, *D_Rmin_* is the minimum dose from the range for the given scenario and *D_Rx_* is the dose prescribed for the given target. The fractional dose deviation was scaled by the prescription dose as target levels varied between primary and secondary targets and between patients. Equation (2) describes the fractional volumetric deviation for the V100% metric.

(2) $$Fractional\;Volumetric\;Deviation=Min\left(V_{fx}-V_{Rmax},V_{Rmin}-V_{fx}\right)\times 100$$

where *V_fx_* is the volume for the given fraction, *V_Rmax_* is the maximum volume from the range for the given scenario and *V_Rmin_* is the minimum volume from the range for the given scenario.

### 2.3. Statistical Analysis

Student’s *t*-tests were performed for the fractional dose deviation between PO and PNN patients for each uncertainty scenario and for PNN patients between the primary and secondary targets. Also, a one-sided paired student’s *t*-test was performed for the fractions within robustness between the 3 mm and 5 mm setup uncertainty scenarios. Statistical significance was determined at the *p* < 0.05 level.

## 3. Results

### 3.1. Patient Demographics and Treatment Characteristics

Six patients with a total of 203 fractions were eligible for analysis. Table 1 summarizes their demographics and clinical characteristics. The patients range from ages 25 to 84 years old with an equal distribution of male and female patients. There was a variety of tumor sites with primarily oropharynx and base of skull locations. All six patients had a primary volume of gross tumor or postoperative tumor bed, and five of the six patients had a secondary volume which included a lower dose nodal or low-risk primary volume that was treated. All volumes were treated with a simultaneous integrated boost technique. The number of fractions ranged from 28 to 48 fractions with both daily and BID fractionation schema. In total, there were two PO patients and four PNN patients. Weight loss was evaluated weekly for five patients; only patients with PNN treatment experienced weight loss at the end of radiation. On average, the PNN patients lost 4.1 kg at the end of treatment, whereas PO patients gained 1.2 kg. Supplementary Table S1 summarizes the patient volumes and organs at risk. Organs at risk were contoured and evaluated at the physician’s discretion given the proximity to the target volumes. Therefore, not all OARS were evaluated for each patient; on average, each OAR was evaluated in 120 fractions (range 66–203).

### 3.2. Daily Shifts

**Figure 1:**
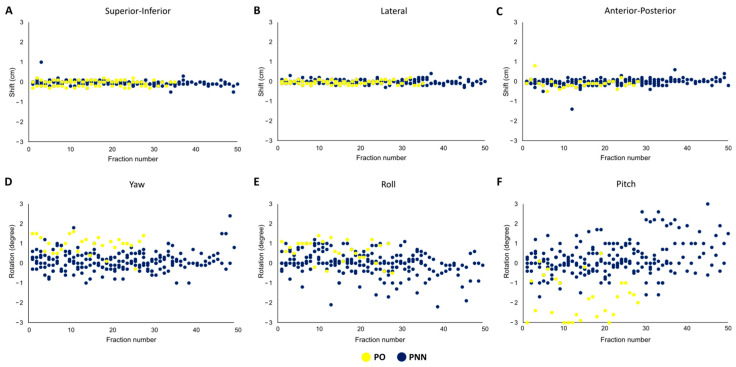
Daily treatment couch shifts. Daily shifts in all six dimensions are described for primary only (PO) and primary and neck nodal (PNN) patients in (**A**) superior–inferior, (**B**) lateral, (**C**) anterior–posterior, (**D**) yaw, (**E**) roll and (**F**) pitch.

We analyzed the magnitude of daily shifts to the treatment couch position from laser to CBCT-based setup in three dimensions and three rotational axes for each fraction for both the PO and PNN patients (Figure 1A–F). The greatest magnitude in the daily shift was found in the transverse dimension (Figure 1F). On average, the pitch rotation was 1.03 cm and 0.65 cm in PO and PNN patients, respectively. The daily shifts were significantly greater for PO patients than for PNN patients (0.44 cm vs. 0.27 cm, respectively, *p* < 0.01) (Supplementary Table S2). We did not find a significant change in magnitude when comparing shifts in the first half vs. the second half of the treatment course (*p* = 0.14).

### 3.3. Fractional Uncertainty

**Figure 2:**
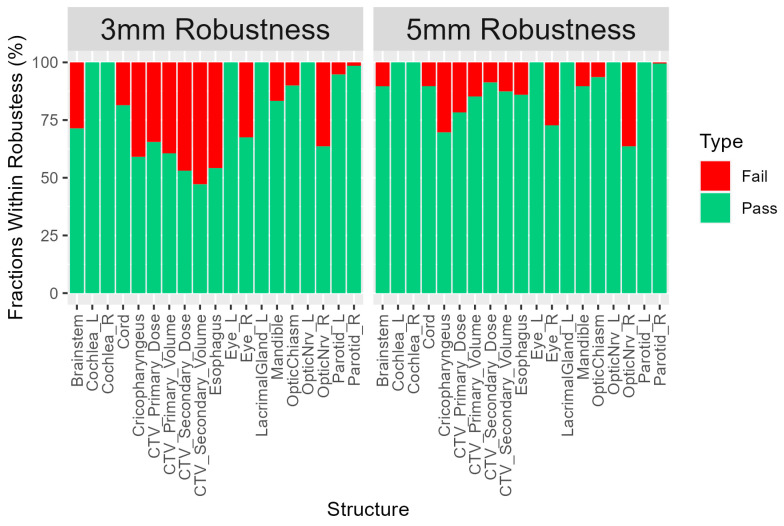
Percentage of fractions within robustness. The fractions within 3 mm and 5 mm setup uncertainty scenarios, both with 3.5% range uncertainty, are shown for all organs at risk and clinical target volumes (CTVs). CTVs were divided into primary and secondary targets. Secondary targets could have multiple nodal levels, which were combined in the secondary volume. CTVs were assessed using D95% designated as Dose and V100% designated as Volume in the figure.

Figure 2 demonstrates the percentage of fractions within robustness for the 3 mm and 5 mm setup uncertainty scenarios. The average percentage of fractions that deviated from the expected uncertainty range for PO and PNN patients was greater using 3 mm as compared to the 5 mm setup uncertainty (13.0% PO and 32.5% PNN at 3 mm vs. 8.6% PO and 11.5% PNN at 5 mm). The percentage of targets outside of the range at 3 mm PO 27.6%, PNN 43.7% and 5 mm PO 19.5%, PNN 12.2%. For the OARs, the mean percentage of fractions which deviated from the expected uncertainty range was 15.10% using 3 mm setup uncertainty as compared to 8.38% of fractions using 5 mm setup uncertainty. The mean percentage of fractions where the OAR dose deviated from the expected uncertainty range was 15.10% of fractions using 3 mm setup uncertainty as compared to 8.38% of fractions using 5 mm setup uncertainty. Fractions in which the OAR dose was greater than the expected dose using 3 mm setup uncertainty were 1.99% on average (range: 0–11.11%) as compared to 0.94% (range 0–7.14%) using 5 mm setup uncertainty.

### 3.4. Fractional Dose Deviation

**Figure 3:**
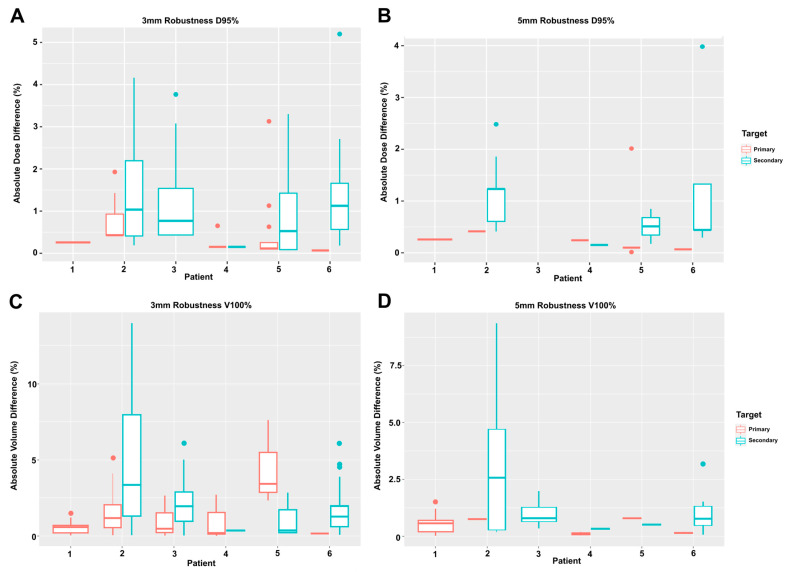
Fractional dose and volume deviation for targets. The fractional dose deviation for D95% using (**A**) 3 mm setup uncertainty and (**B**) 5 mm setup uncertainty. The fractional volume deviation for V100% using (**C**) 3 mm setup uncertainty and (**D**) 5 mm setup uncertainty The primary targets are shown in red and nodal targets in blue. Patients with no error bar indicate all fractions meet the dose or volume constraint.

For patients who had fractions outside of the uncertainty ranges, we calculated the fractional dose and volumetric deviation to determine the distance that the given fraction was outside the D95% and V100% range from the uncertainty scenarios. Supplementary Figure S1 demonstrates the DVH deviation per fraction for each patient. Figure 3A,B shows the fractional dose difference for the two setup scenarios for the primary and secondary targets for each patient. It clearly demonstrates the larger dose difference for secondary targets. Using 3 mm setup uncertainty, 15.27% of the PO and 45.53% of PNN fractions were below the prescribed D95%. Using 5 mm setup uncertainty, 1.48% of PO and 7.26% of PNN fractions were below the prescribed D95%. The average fractional dose deviation at 3 mm setup uncertainty was 0.22% (range: 0.15–0.65%) for PO patients and 1.02% (range: 0.07–5.20%) for PNN patients and at 5 mm setup uncertainty was 0.24% (range: 0.15–0.26%) for PO patients and 0.52% (range: 0.01–3.98%) for PNN patients. The average fractional dose deviation was statistically different between PO and PNN patients at 3 mm (*p* = 0.01) but not 5 mm setup uncertainty (*p* = 0.27). For PNN patients, the average fractional dose deviation at 3 mm was 0.35% and 1.25% for primary and secondary targets, respectively, and 0.13% and 1.00% at 5 mm setup uncertainty for primary and secondary targets, respectively. For both scenarios, the difference was statistically significant (3 mm: *p* < 0.01; 5 mm: *p* < 0.01).

### 3.5. Fractional Volume Deviation

Figure 3C,D shows the fractional volumetric difference between the two setup scenarios for the primary and secondary targets for each patient. Using 3 mm setup uncertainty, 25.62% of the PO and 52.51% of PNN fractions were below the prescribed V100%. Using 5 mm setup uncertainty, 0.99% of PO and 10.89% of PNN fractions were below the prescribed V100%. The average fractional volumetric deviation at 3 mm setup uncertainty was 0.68% (range: 0.01–2.71%) for PO patients and 2.10% (range: 0.02–13.96%) for PNN patients and at 5 mm setup uncertainty was 0.36% (range: 0.01–1.51%) for PO patients and 1.80% (range: 0.07–9.35%) for PNN patients. The average fractional volumetric deviation was statistically different between PO and PNN patients for both scenarios (3 mm: *p* < 0.01; 5 mm: *p* < 0.01). For PNN patients, the average fractional volumetric deviation at 3 mm was 1.50% and 2.20% for primary and secondary targets, respectively, and 0.46% and 1.90% at 5 mm setup uncertainty for primary and secondary targets, respectively. The fractional volumetric deviation between primary and secondary targets was only statistically significant for 3 mm setup uncertainty (*p* = 0.05) and was not significant for 5 mm setup uncertainty (*p* = 0.20).

## 4. Discussion

Our evaluation of daily fractional dose in head and neck patients treated with IMPT describes two significant findings: (1) PO patients have greater daily treatment couch shifts than PNN patients. This is likely due to the smaller volume that needs to be covered in PO disease, giving more flexibility in adjusting to daily setup differences using CBCT. This was most significant for pitch rotations on the transverse plane but also applied to all other planes except the lateral shifts. (2) The percentage of fractions that deviated outside of the predicted robustness scenarios was significantly higher using 3 mm setup robustness as compared to 5 mm for both PO and PNN plans. However, the calculated fractional deviation using both 3 and 5 mm setup uncertainty was not clinically relevant for PO patients. Although we found a few large variations in the daily fractional dose and volume, the majority of fractions were within robustness, and the average fractional D95% deviation for fractions that were outside robustness for PNN patients over the course of treatment for both the primary and secondary targets was up to 1% and absolute volumetric V100% deviation was up to 2% using 3 mm setup uncertainty.

Unlike photon planning processes where the PTV is a uniform expansion from the clinical planning target, IMPT planning takes into account setup and range uncertainty directly and optimizes the plan to deliver uniform doses across the CTV under nominal and robust scenarios [21]. However, there have been few approaches to better define the inter- and intra-fractional variations, which can have significant consequences on the delivered dose to the patient. Although there are no guidelines for an acceptable fractional dose deviation based on setup uncertainty, we can extrapolate from ICRU 83 recommendations for IMRT planning, where a near minimum dose of D98% is an acceptable planning aim [22]. Therefore, we believe that the up to 2% average fractional dose deviation from the robustness calculation using a 3 mm setup uncertainty is acceptable.

Using synthetic CTs, we have developed an approach to validate simulated robustness criteria [8,10,11] and better understand the significance of inter-fractional dose changes on actual patient dose accuracy. Currently, there are no guidelines for acceptance of worst-case robustness scenarios as alignment deviation is believed to be a random occurrence [21]; therefore, repeated daily shift deviation in one direction would be uncommon. We have found that there can be a large magnitude in daily treatment couch shifts, reiterating the need for daily CBCT imaging. In addition, small rotational deviations are more meaningful in the displacement of elongated targets, such as nodal treatment in head and neck patients; therefore, smaller alignment changes would be made. We similarly found a smaller magnitude of shifts in PNN vs. PO setup using image guidance, likely due to on-treatment shifts covering a much larger volume.

Daily setup and plan dose verification demonstrates the practical application of using synthetic CT to understand dose distribution on patients undergoing IMPT. This has a number of implications, particularly in head and neck cancer, where adaptive planning is common given changes in patient anatomy over the course of treatment [23]. The current clinical practice uses quality assurance CT imaging to evaluate the need for replan; however, this process could introduce inaccuracies, as patient setup may not replicate the setup on the treatment machine. Clinical workflows using synthetic CT derived from on-treatment CBCT would improve how physicians assess dose distribution at multiple timepoints during treatment and anticipate the need for adaptive planning [24]. In addition, synthetic CT imaging could be used for adaptive planning and removes the need for repeating the simulation CT, which results in additional radiation exposure [16,25].

There are several limitations to our study, including the small cohort and the limitation to a single institution and treatment planning system. For example, immobilization devices can differ on inter- and intra-fractional displacement [26]; this is a systematic error that should be relatively controlled for in our dataset, as all patients used the same immobilization device. It is important to collaborate with other institutions in future studies to demonstrate the reproducibility of our findings. Our daily robustness analysis relies on deformable image registration to the planning CT, which could theoretically affect dosimetric accuracy. Comparing synthetic CT to quality assurance CT dose distribution in the upper cervical spine, our group has found that the gamma map (γ2%/2 mm) pass rates were 99.0% [16]. Similarly, other groups have found accurate proton dosimetric evaluation using deformable image registration [17]. We did not assess plan robustness in patients who required an adaptive plan. We hypothesized that this cohort of patients was all found to have significant changes in dose distribution on quality assurance CTs, prompting plan adaptation, and therefore, it was not the ideal dataset for this analysis. However, despite these limitations, we believe that these practical planning considerations can significantly contribute to plan quality assurance.

## 5. Conclusions

Treatment planning for intensity-modulated proton therapy in head and neck treatment requires validation of the actual received dose, given inherent uncertainties within the proton planning and delivery process. Based on our analysis, we suggest the following recommendations: (1) Patients with PO disease should use 3 mm setup uncertainty when using daily CBCT and (2) patients with PNN disease being planned for IMPT can use 3 mm setup uncertainty, with the understanding that actual fractional deviation may be up to 2% greater than the robustness calculation. Given the importance of dose falloff for critical OARS, proton planning needs to balance the goals of dose accuracy with OAR sparing. Our study validates 3 mm robustness optimization as an acceptable simulation of dose delivered with minimal inaccuracy in actual dose delivery.

## Figures and Tables

**Table 1:** Patient and treatment characteristics.

Cases (*n* = 6)	
Age at treatment (yrs), mean (SD)	50.5 (22.7)
Sex:	
Male	3 (50.0%)
Female	3 (50.0%)
Location:	
Sinonasal	2 (33.3%)
Tonsil	2 (33.3%)
Schwannoma	1 (23.8%)
Salivary Gland	1 (23.8%)
T Stage:	
T1	1 (23.8%)
T2	1 (23.8%)
T3	1 (23.8%)
T4	3 (50.0%)
N Stage:	
N0	2 (33.3%)
N1	2 (33.3%)
N2	2 (33.3%)
Primary prescription (Gy), mean (SD)	67.1 (8.7)
Secondary prescription (Gy), mean (SD)	63.0 (2.8)
Weight loss (kg), mean (SD)	−2.0 (3.8)

## Data Availability

The data presented in this study are available on request from the corresponding author.

## Supplementary Materials

The following supporting information can be downloaded at https://www.mdpi.com/article/10.3390/cancers15143719/s1, Table S1: Per patient organ fractionation; Table S2: Treatment shifts (cm). Supplementary Figure S1: Patient fractional DVH: Patient 1 (A), Patient 2 (B–D), Patient 3 (E–G), Patient 4 (H–K), Patient 5 (L–M) and Patient 6 (N–P). Blue dashed lines indicate robustness bands of 3.5% and 5 mm setup uncertainty and black lines indicate robustness bands of 3.5% and 5 mm setup uncertainty.
