# Supplementary material for: Daily Head and Neck Treatment Assessment for Optimal Proton Therapy Planning Robustness
Source: Cancers (Basel). 2023 Jul 22;15(14):3719. doi: 10.3390/cancers15143719 (PMC10378634; doi:10.3390/cancers15143719)
Supplement: Supplementary file 1 [file cancers-15-03719-s001.zip › cancers-2418695-supplementary tables.pdf]

supplementary material

Table S1: Per Patient Organ Fractionation

ROI	# Patients	# Fractions
CTV_Primary	6	203
CTV_Secondary	5	358
Brainstem	6	203
Cord	3	97
Mandible	4	126
Parotid_L	5	173
Parotid_R	5	175
Cochlea_L	4	140
Cochlea_R	3	112
Cricopharyngeus	2	66
Esophagus	3	107
Eye_L	2	77
Eye_R	2	77
OpticChiasm	3	110
OpticNrv_L	3	110
OpticNrv_R	3	110

Table S2: Treatment Shifts (cm)

Shift dimension	PO	PNN	P-value
Superior/Inferior	0.12	0.08	0.01
Lateral	0.09	0.08	0.27
Anterior/Posterior	0.17	0.09	<0.01
Coronal	0.62	0.36	<0.01
Sagittal	0.63	0.39	<0.01
Transverse	1.03	0.63	<0.01
Average	0.44	0.27	<0.01
