# Supplementary figures and images for: Daily Head and Neck Treatment Assessment for Optimal Proton Therapy Planning Robustness
Source: Cancers (Basel). 2023 Jul 22;15(14):3719. doi: 10.3390/cancers15143719 (PMC10378634; doi:10.3390/cancers15143719)

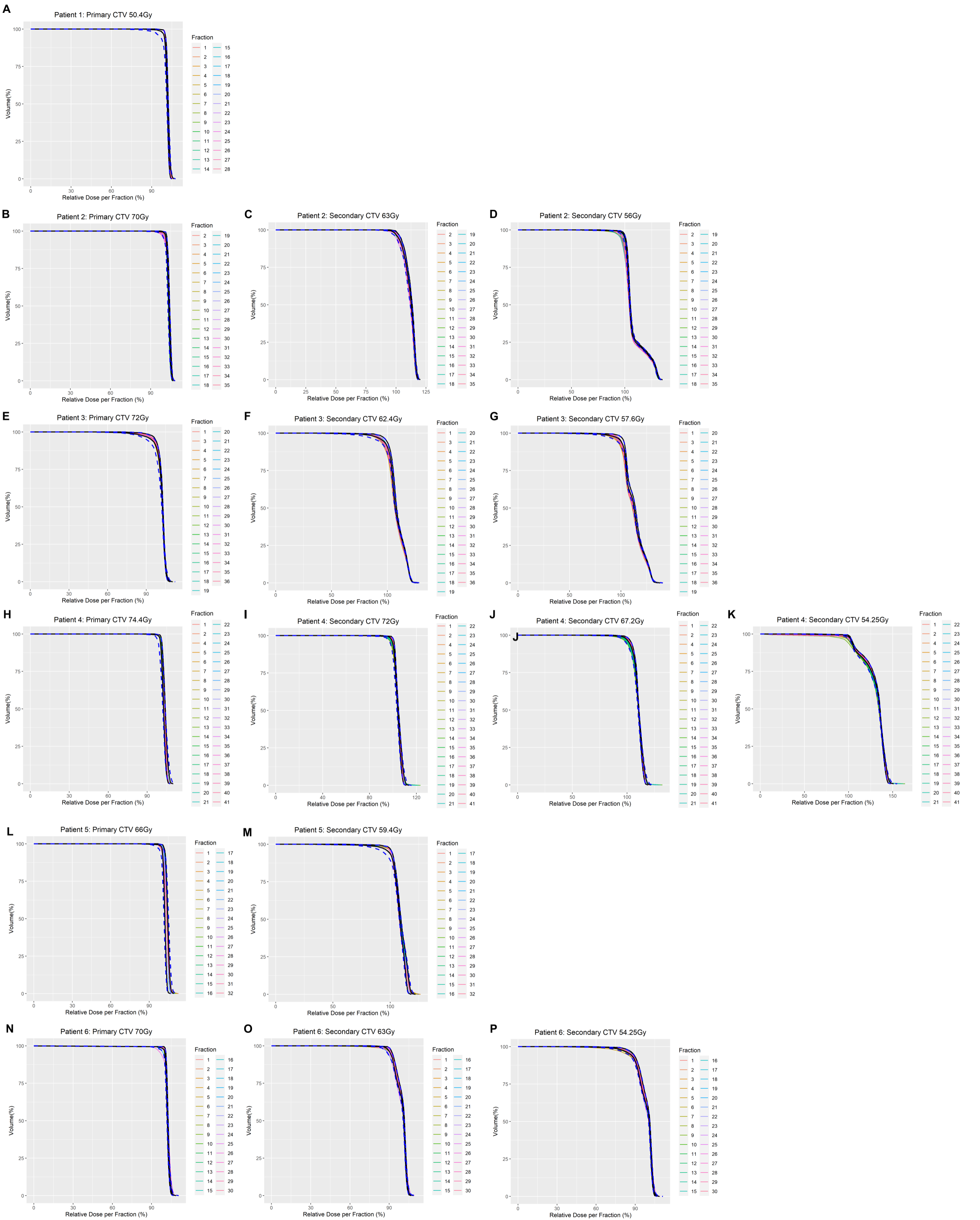

Supplement: Supplementary file 1 [file cancers-15-03719-s001.zip › Supplementary Figure S1.pdf]
